# The Application of Aptamer and Research Progress in Liver Disease

**DOI:** 10.1007/s12033-023-01030-4

**Published:** 2024-02-02

**Authors:** Cheng Xu, Yong Tan, Li-Ye Zhang, Xiao-Jie Luo, Jiang-Feng Wu, Lan Ma, Fei Deng

**Affiliations:** 1 Hubei Key Laboratory of Tumor Microenvironment and Immunotherapy, China Three Gorges University, Yichang, China; 2 College of Basic Medical Science, China Three Gorges University, Yichang, 443002 Hubei China; 3 Institute of Organ Fibrosis and Targeted Drug Delivery, China Three Gorges University, Yichang, China; 4 Hubei Selenium and Human Health Institute, The Central Hospital of Enshi Tujia and Miao Autonomous Prefecture, Enshi, Hubei China; 5 Department of Oncology, The Second People’s Hospital of China Three Gorges University, Yichang, 443000 China

**Keywords:** Aptamer, Patent, Liver diseases, Biomarker, Drug delivery

## Abstract

**Abstract:**

Aptamers, as a kind of small-molecule nucleic acid, have attracted much attention since their discovery. Compared with biological reagents such as antibodies, aptamers have the advantages of small molecular weight, low immunogenicity, low cost, and easy modification. At present, aptamers are mainly used in disease biomarker discovery, disease diagnosis, treatment, and targeted drug delivery vectors. In the process of screening and optimizing aptamers, it is found that there are still many problems need to be solved such as the design of the library, optimization of screening conditions, the truncation of screened aptamer, and the stability and toxicity of the aptamer. In recent years, the incidence of liver-related diseases is increasing year by year and the treatment measures are relatively lacking, which has attracted the people’s attention in the application of aptamers in liver diseases. This article mainly summarizes the research status of aptamers in disease diagnosis and treatment, especially focusing on the application of aptamers in liver diseases, showing the crucial significance of aptamers in the diagnosis and treatment of liver diseases, and the use of Discovery Studio software to find the binding target and sequence of aptamers, and explore their possible interaction sites.

**Graphical Abstract:**

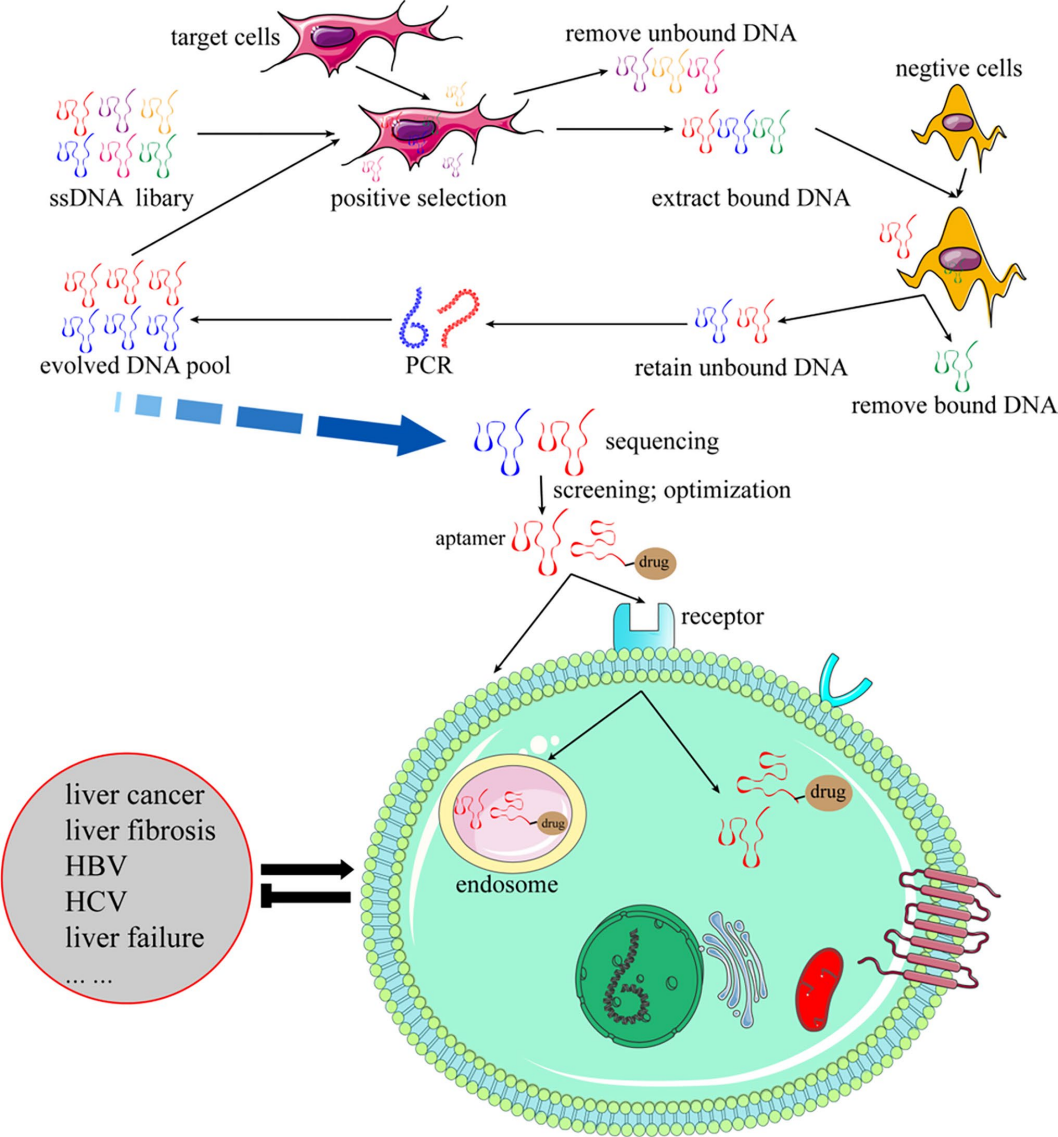

**Supplementary Information:**

The online version contains supplementary material available at 10.1007/s12033-023-01030-4.

## Introduction

Nucleic acid aptamer also known as aptamer is a single-stranded DNA or RNA molecule, which is 20 to 100 nt in length. They were first isolated in the early 1990s by Tuerk and Gold using the Systematic Evolution of Ligands by Exponential Enrichment (SELEX) technique [1, 2]. Since its discovery, many aptamers have been screened [3, 4], compared with antibodies, aptamers have the unique advantages of low molecular weight, low cost, easy chemical modification, non-toxic, low immunogenicity, easy to penetrate tissue barrier, and multi-target sites (amino acids, peptides, proteins [4–14], antibiotics [15], cells [16–19], viruses [20–23], bacteria [24, 25], ions [26], etc.), so it has been paid more and more attention. Different diseases have different specific targets, even the same disease also has a number of different targets, such as in the progress of liver fibrosis, TLR4, inflammatory cytokines, and TGF-beta are associated with fibrosis process, and only specific monoclonal antibody against a target cannot satisfy all the targets [27], whereas aptamers could overcome this difficulty.

Some diseases of liver, such as tumors and liver fibrosis, usually have no obvious clinical symptoms in the early stage, and need to rely on the gold standard of liver biopsy for diagnosis [28]. However, due to its high cost and aggressiveness, few patients are willing to undergo liver tissue biopsy at an early stage. Therefore, it is necessary to find a diagnostic method with low invasiveness and high specificity. Previous experimental studies have demonstrated the multifaceted potential of aptamers in both liver disease diagnosis and targeted drug delivery [29], thereby offering a novel approach for the diagnosis and treatment of liver-related diseases [30].

However, aptamers have some limitations and uncertainties in the diagnosis and treatment of diseases. A series of partial potential biomarkers have been obtained through modern genomics, proteomics, single-cell sequencing analysis, and other technologies, but they are rarely applied in clinical diagnosis and treatment due to their low specificity. Though, screening through some biomarkers have obtained corresponding aptamers through screening, such as Sgc8 targeting PTK7 [31], Pegaptanib targeting VEGF [32], and AS1411 targeting nucleolin [33], additionally, a series of aptamers and corresponding target proteins have been identified so far. Due to the difference between protein purification and its original state, the mechanism of action of aptamers and target proteins has not been fully confirmed. At the same time, many aptamers are obtained by cell-SELEX technology screening, and the target is the whole cell. The specific molecular targets and binding methods are still unclear. However, if these limitations can be solved, the application of aptamers will make breakthrough progress.

So, the article summarizes the recent progress and current situation of aptamers in the diagnosis and treatment of diseases, especially in liver disease, to provide advice for screening and application of aptamers in the future.

## Update Patented Aptamer (1990–Present)

### Recent Progress and Status of Aptamer

Aptamers have been widely concerned in various fields because of their high affinity, strong specificity, and strong stability in binding to targets [34]. Since the first aptamer was discovered, a large number of aptamers have been screened and optimized, and aptamers have promoted the development of basic research on biosensors, drug delivery systems, and disease diagnosis systems [35]. At present, the application of aptamers in medicine is mainly in the diagnosis and treatment of cancers. For example, the aptamer drug supported by the US FDA on the market at present is Macugen (Pegaptanib), an RNA aptamer targeting vascular endothelial growth factor (VEGF), which is applied to the treatment of eczema-related macular degeneration [36]. In SELEX screening, the performance of aptamers such as those targeting proteins may not be better than those obtained by targeting cells because the target molecules in cell-SELEX are closer to the original state of the in vivo environment [37].

**Fig. 1:**
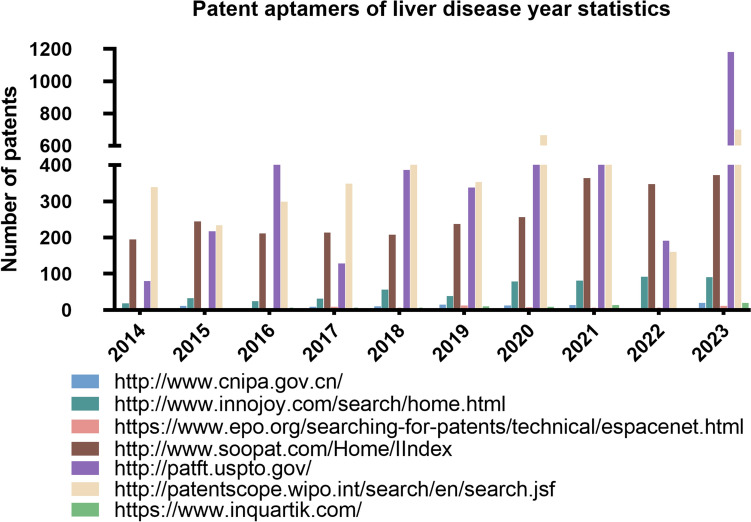
The number of patent aptamers for liver diseases since 2014. keywords included: “aptamers,” “liver disease”

Due to the simplicity of aptamer screening procedures and principles, a significant number of aptamers have been screened and patented. However, there is a limited availability of aptamers for biosensors, diagnostic probes, and therapeutic reagents, possibly due to challenges in post-screening modification and optimization [38]. By conducting searches on patent websites, one can gain insights into the quantity and development trends of aptamers in recent years. The results are depicted in Fig. 1, which indicates a gradual increase in the annual number of patent applications for liver disease-specific aptamers, highlighting their promising application prospects. Figure S1 of supply materials also presents an overview of the total number of aptamer patents since 2014.

### The Aptamers are Currently in Clinical Trials

The aptamer can be optimized by truncating their sequences to reduce molecular weight and improve binding affinity, and can also be combined with high molecular weight fragments such as polyethylene glycol [39], cholesterol [40], protein [41], or nanomaterials [42] to prolong renal excretion. After sequence truncation and conjugation, modification at nucleotide ribose 2 position with –O–Me, –NH_2_, –F, etc., can prevent nuclease degradation [43]. Aptamer research is not going well in ongoing clinical trials, for example, E10030 inhibits fibroblast proliferation by blocking platelet-derived growth factor B chain (PDGF-B), and was declared a failure in phase III in august 2017, because E10030 combined with Ranibizumab did not prove to be more effective than Ranibizumab alone. Another example is the use of aptamer REG1 for anticoagulation therapy which was terminated in the trial due to severe allergic reactions [33]. Despite these difficulties, a number of aptamers are currently in clinical trials for the treatment of ocular diseases, thrombotic and vascular diseases, and cancers [44]. In terms of drug delivery, aptamers can replace other transporters for targeted delivery of drugs. For example, an aptamer specific for transferrin receptor CD71 (TfR) can replace transferrin and play an alternative role in cellular imaging or targeted drug delivery [45]. It can be used as a diagnostic reagent for biosensors by hybridization with DNA, RNA, peptides, or nanomaterials for specific targeting and imaging [46]. Clinical trials of aptamers can be found on https://clinicaltrials.gov/, a total of 44 aptamers and drug test the aptamer-related projects, the apatamers which have entered clinical trials included 12. Table 1 summarizes all aptamers that have entered clinical trials, including the diseases they have treated, the targets of action, and the clinical stages of investigation.

**Table 1:** Aptamers are in clinical trials

Name	Target	Disease	Extension	Clinical Trial	References
E10030	PDGF	AMD	Platelet-derived growth factor B chain (PDGF-B)	Phase 2	[47, 48]
Pegaptanib	VEGF	AMD	High-affinity VEGF receptor-binding nucleic acid ligands and inhibitors of VEGF function	Therapy	[31]
EYE001	VEGF	AMD	Anti-VEGF	Phase 2/phase 3	[49]
ARC1905	C5	AMD	Treatment and prevention of eye diseases	Phase 1	[50]
NOX-H94	hepcidin	Anemia/renal disease	Hepcidin-specific approach to the treatment of anemia of chronic disease	Phase 1/phase 2	[51]
NOX-E36	CCL2	Type 2 diabetes/diabetic nephropathy	Monocyte chemoattractant protein-1 (MCP-1)	Phase 1	[52, 53]
NOX-A12	SDF-1	Multiple myeloma chronic stromal lymphocytic leukemia	Cell-derived factor- 1	Phase 1	[54]
BAX499	TFPI	Hemophilia	Tissue factor pathway inhibitor (TFPI)	Phase 2	[55]
AS1411	nucleolin	Acute myeloid leukemia	Antiproliferative activity of G-rich oligonucleotides and method of aptamer binding to nucleolin	Phase 2	[56]
REG1	coagulation factor IXa	Coronary artery disease/ ACS	Administration of the REG1 anticoagulation system	Phase 1	[57]
ARC1779	VWF	Thrombosis	Aptamers binding to VWF and their use as thrombotic disease therapeutics	Phase 2	[58]
Sgc8	PTK7	Colorectal cancer	Specific ligand of PTK7, chemotherapeutic drug delivery	Early phase 1	[59]

*PDGF-B* platelet-derived growth factor B chain, *AMD* age-related macular degeneration, *VEGF* vascular endothelial growth factor, *CCL2* C–C motif ligand 2, *SDF-1* stromal cell-derived factor-1; *TFPI* tissue factor pathway inhibitor, *VWF* von Willebrand factor, *PTK7* protein tyrosine kinase 7

#### Discussion on the Use of Aptamers as Therapeutic Drugs

When applied to biological agents, aptamers are often used as therapeutic agents to regulate biological pathways and intervene in a variety of diseases [60]. The treatment of neovascular-related macular degeneration by intravitreal injection of aptamer E10030 in combination with Ranibizumab has proven successful in safety and efficacy despite failure in phase III clinical trials [47, 48]. The aptamer Pegaptanib, as an anti-VEGF antagonist, has been approved by the US FDA for intravitreal injection to block VEGF and thus treat AMD [61, 62], the first successful treatment for wet AMD. Although its use and results are not perfect, its benefits in treating AMD far outweighs its risks [63]. EYE001, a VEGF-targeting aptamers, has been shown in preclinical and clinical studies to improve vascular permeability and ocular neovascularization, with no significant side effects in a single-dose intravitreal injection in phase Ia clinical trials [64]. ARC1905, an aptamer that inhibits complement factor C5 to prevent terminal fragment structure by screening, is mainly used in the treatment of age-related macular degeneration, and a phase I study is currently under way to evaluate its safety and efficacy in combination with Lucentis [65, 66].

NOX-H94 is a structural mirror aptamer, which specifically binds hepcidin (Hep) and regulates chronic inflammatory anemia by blocking the biological function of Hep. As a non-natural mirror aptamer, it is not recognized by nuclease and immune system, and has certain safety [67]. NOX-E36 has a length of 40 nucleotides and can specifically bind to the proinflammatory chemokine C–C motif ligand 2 (CCL2). NOX-E36 is mainly used in the treatment of type 2 diabetes and has performed well in phase I clinical trials. It has an inhibitory effect on CCL2 without activating the innate immune system, which can be used as a new targeted therapy for diabetic kidney injury [51]. NOX-A12 is a novel pegylated oligonucleotide that specifically binds to the chemokine SDF-1 and is mainly used in the treatment of chronic lymphocytic leukemia. At present, its phase II clinical results show good safety and efficacy, and it can be used in combination with other targeted drugs to enhance efficacy and reduce drug side effects [68]. BAX499, formerly known as ARC19499, is an anti-tissue factor pathway inhibitor (TFPI) inhibitor. It mainly combines with TFPI to inhibit TFPI-mediated tissue factor pathway and shorten whole blood coagulation time. Furthermore, the bleeding of hemophilia patients is inhibited, so as to achieve the purpose of treatment [69]. AS1411 is an aptamer composed of 26 G-rich nucleotides, which can form a G-quadruplex structure. It is mainly used in cancer-targeted drug delivery, and has also been developed as a targeted drug or probe with high safety. At present, it is mainly connected with nanoparticles for targeted therapy or imaging of cancer [70]. As an anticoagulant, aptamer REG1 performs well against platelet thromboembolism, especially in cardiovascular disease. However, as an anticoagulant, its pharmacodynamics and immunogenicity are unpredictable, and it was eventually discontinued in clinical trials due to severe allergic reactions. Aptamer ARC1779 primarily inhibits von Willebrand factor (VWF), which causes thrombocytopenia in thrombosis. The current experimental results show that ARC1779 is well tolerated and safe, and has a good recovery effect on organ failure caused by thrombocytopenic purpura [71]. In the future, aptamers and their derived sensors and probes will provide multiple options for the diagnosis and treatment of diseases [44].

Cost-effectiveness analysis should also be considered in the application of aptamers. Using Pegaptanib as an exemplar, the FDA approved aptamer drug for AMD. AMD refers to age-related structural changes in the macular area of the eye, predominantly affecting individuals over 50 years old and representing a significant cause of blindness among older adults. Pegaptanib is a 28-base RNA aptamer employed as a vascular endothelial growth factor antagonist in AMD treatment. Current therapeutic options for AMD encompass ranibizumab, Pegaptanib, bevacizumab, and PDT with verteporfin. Studies have demonstrated that ranibizumab is considered the most cost-effective among approved regimens for treating AMD [72]. However, UK-based analysis revealed that Pegaptanib exhibited similar cost-effectiveness compared to best supportive care over a decade-long timeframe [73]. Nevertheless, it should be noted that the cost-effectiveness of Pegaptanib varied considerably depending on disease stage and time horizon [72, 74, 75].

#### Discussion on the Use of Aptamers as Drug Delivery Systems

Aptamer can be used not only as a drug directly but also as a drug carrier to deliver drugs directly to cells by coupling drugs such as siRNA, decoy ODN, and micro-RNA [76]. Existing RNA, DNA aptamers, and siRNAs are covalently or non-covalently combined to form chimeras, and modified, such as polyethylene glycol (PEG), to prolong degradation time and improve their bioavailability in vivo [77], while its side effects are significantly lower than those of chemotherapy and radiotherapy drugs [60, 65]. The aptamer Sgc8, which is currently in clinical trials, can target PTK7 as a potential drug for the treatment of malignant hematological diseases, solid tumors, and other diseases [78]. However, the development of aptamers has been questioned due to many setbacks in clinical trials [79]. One of the important issues is the synthesis and modification of aptamers. The synthesis of long sequence aptamers, especially RNA, is very difficult, and some special modifications can also increase the cost, limiting the clinical application of aptamers [76]. With the development of computer technology, molecular simulation technology can realize the prediction of chemical synthesis, modification, and structure. It is believed that the application of aptamers will make greater progress in the near future.

## Application of Aptamer in Liver Disease

### Liver Disease-Related Biomarkers

Liver is the main metabolic organ of human body. Liver diseases are usually triggered by the death of hepatocytes and progresses to hepatitis, fibrosis, cirrhosis, and liver cancer. At present, liver diseases mainly include acute liver diseases caused by food poisoning and infection, and chronic liver diseases caused by viral infection, fatty liver, alcoholic hepatitis, etc. [80]. In different liver injuries, there may appear many different biomarkers. Some of these specific markers play an important role in the diagnosis and targeted therapy of liver disease. ALT and AST are the most common biomarkers with significantly increased expression after liver injury, but lack specificity [81]. Other common biomarkers, such as K18, HMGB1, and micro-RNAs, also sensitively reflect liver injury. Moreover, combined with complementary markers, it can be used as a commonly used diagnostic index to effectively diagnose liver diseases caused by various factors [80]. Table S1 (shown in supply materials) summarizes the current biomarkers in the liver diseases process and Table 2 concludes the aptamers of known biomarkers obtained by screening which is used in liver diseases. But at present, maybe there are many studies on specific markers of liver cancer, so the current research on the applications of aptamers in the diagnosis and treatment of liver diseases is mainly aimed at liver cancers, such as develop a label-free microcantilever array aptasensor to detect HepG2 cells and to provide a simple method for the detection of liver cancer cells [82], use an aptamer-nanotrain to deliver doxorubicin selectively to liver cancer cells [83]. As for other kinds of liver diseases, TNF-α-targeting aptamer can attenuate the degree of hepatocyte damage and potentiate early regeneration of the liver tissues in TNF-α-mediated acute liver failure [30], and aptamer-functionalized ultrasound nanobubbles with resveratrol and ultra-small copper-based nanoparticles can treat non-alcoholic fatty liver diseases [84].

**Table 2:** Aptamers are used in liver diseases

Name	Target	Disease	Subject	Application	References
TLS11a	HepG2	HCC	Specific detection of hepatocellular carcinoma derived	Diagnose/therapy	[85–87]
sLex-AP	sLex	HCC	Efficient capture of HCC CTCs	Diagnose	[88]
AFB1-Apt	AFB1	Liver cancer	Small doses cause liver cancer	Diagnose	[89]
SL_2_-B	VEGF165	Liver cancer	SL_2_-B aptamer could specifically recognize HepG2 liver cancer	Diagnose	[90]
Slow-off/fast-off aptamer	DKK1	HCC	Early diagnosis of HCC	Diagnose	[91]
CD105-Apt	Surface endoglin	HCC	Hepatocellular carcinoma targeting nano-probe	Diagnose	[92]
mEND	CD105	HCC	Targeted nanometer probes enhance MRI efficiency	Diagnose	[93]
AP613-1	GPC3	HCC	Diagnosis of early HCC	Diagnose	[94]
Ep1	EpCAM	Liver cancer	Targeted drug delivery and identification of cancer cells as imaging agents	Delivery/therapy	[95]
EpDT3	EpCAM	Liver cancer	Carries therapeutic drugs to suppress tumors	Delivery	[96]
ST21	SK-Hep-1 cells	HCC	Conjugated and delivery drugs to overcome HCC	Delivery	[97]
TLS1c	MEAR cells	Liver cancer	Detection of CTCs, early cancer diagnosis	Diagnose	[98]
AP273	AFP	HCC	Diagnosis of cancer	Diagnose	[99]
SOMAmer	GPC3	HCC	Quantitative GPC3 in HCC patients	Diagnose	[100]
AS1411	Nucleolin	HCC	Modified AS1411-aptamers may have therapeutic potential as a novel targeted therapy for HCC	Therapy	[101, 102]
GT75	eEF1A1	HCC	Effectively reducing HCC cells viability in a dose and time dependent fashion	Therapy	[103]
LCN2_apta2/LCN2_apta4	LNC2	HCC	Diagnosing HCC and potential applicability to the point-of-care testing (POCT) system	Diagnose	[104]
APT	OPN	HCC	APT targeting OPN significantly reduces the growth of HCC tumor	Therapy	[105]
BC15	hnRNP A1	Liver cancer	Inhibit effect on the proliferation of hepatoma cells	Therapy	[105]
RNV-L7	LDL-R	Liver cancer	LDL-R was highly expressed in huh-7 cells with specific binding	Delivery	[106]
CL-4RNV616	EGFR	Liver cancer	Effectively recognized and inhibited the proliferation of EGFR-positive Huh-7 liver cancer	Therapy	[107]
LY-1	HCC cell	Liver cancer	Target and suppress tumor	Therapy	[108]
aptTNF-α	TNF-α	Liver failure	Reduce the damage degree of liver cells after acute injury and enhance the early regeneration of liver tissue	Therapy	[30]
Apt-PBMCs	CRP	Liver failure	In vitro tracing of CRP secretion site	Diagnose	[109]
T18_1_3	TGFβ-1	Liver fibrosis	An important tool to study fibrosis of the liver	Therapy	[110]
Aptamer-20	IGFIIR	Liver fibrosis	IGFIIR-specific aptamer used as a targeting ligand for the treatment and diagnosis of liver fibrosis	Diagnose/therapy	[111]
HBsAg-specific aptamer	HBsAg	HBV	Highly sensitive detection of HBsAg	Diagnose	[112]
NS2-2/NS2-3	NS2	HCV	Bind to the NS2 and disrupt the interaction of NS2 with NS5A protein	Therapy	[113]
NS5B aptamer	NS5B	HCV	Inhibited HCV RNA replication	Therapy	[114]
RAGE-aptamer	RAGE	Melanoma liver metastasis	Inhibits tumor angiogenesis and inhibits melanoma growth and liver metastasis	Therapy	[115]

*AFB1* Aflatoxin B1, *DKK1* Dickkopf-1, *EpCAM* Epithelial Cell Adhesion Molecule, *AFP* Alpha-fetoprotein, *GPC3* Glypican 3, *eEF1A1* Eukaryotic elongation factor 1A1, *GP73* Golgi protein 73, *LNC2* Lipocalin-2, *OPN* Osteopontin, *hnRNP A1* Heterogeneous nuclear ribonucleoproteins, *LDL-R* Low-Density Lipoprotein-Cholesterol, *EGFR* Epidermal growth factor receptor, *TNF-α* Tumor Necrosis Factor α, *CRP* C-reactive protein, *IGFIIR* Insulin-like growth factor II receptor, *NS2* non-structural protein 2, *NS5B* Non-structural protein 5B

### Aptamers are Used in the Diagnosis and Therapy of Liver Cancer

Cancer may not show symptoms in its early stages, which prevents timely diagnosis and treatment of cancer at an early stage. If in the early stages of cancer, biomarkers have emerged, then it is of great significance to screen aptamers targeting biomarkers as early tumor-specific diagnostic and therapeutic reagents. As probes, aptamers have better specificity and sensitivity than AFP, CEA, and other biomarkers for diagnosis [116]. Liver cancer is one of the five most common cancers in the world, among which HCC is the most common [117]. The traditional treatment strategy for HCC is surgery, but the recurrence rate is as high as 70%. Liver cancer stem cells (LCSC) have been found to have a great relationship with the growth, transformation, and metastasis of liver cancer [118]. Therefore, the main treatment direction is to find specific markers of LCSC as therapeutic targets for targeted drugs. Table 2 presents a comprehensive compilation of 22 aptamers, which have been identified as valuable diagnostic, therapeutic, and targeted delivery tools for liver cancer. Among them, the aptamer CL-4RNV616 specifically targets the epidermal growth factor receptor (EGFR), which is highly expressed in many cancers and is considered a prognostic indicator of cancer [119]. The specific recognition of EGFR protein in tumor cells can be used as a targeted probe for early cancer detection, but the binding ability of MDA-MB-231 breast cancer cells, hum-7 liver cancer cells, and U87MG glioma cells suggests non-specific cell recognition [107].

### Aptamers are Used in Early Diagnosis of Liver Fibrosis

Liver fibrosis is an intermediate process of liver injury and inflammation caused by hepatitis virus infection, alcohol abuse, immune response, drug and chemical damage, and then develops into chronic progressive liver disease. During liver injury, inflammation, and repair, hepatic stellate cells (HSCs) located in the perisinusoidal space are activated and transformed into myofibroblasts (MFCS). MFCS produce large amounts of collagen, mainly extracellular matrix (ECM), resulting in liver fibrosis [120, 121]. Various cytokines and related signaling pathways in the development of liver fibrosis, as well as the pathways of stellate cell clearance (apoptosis, senescence, recovery to inactivation), have been clearly elucidated. Liver fibrosis eventually develops into cirrhosis and liver cancer. Liver fibrosis has been shown to be reversible, so treatment of liver fibrosis can effectively prevent cirrhosis and liver cancer [122]. At present, RNA interference (RNAi) has been studied for the treatment of liver fibrosis, but its targeting and effectiveness into the body are poor, so it is necessary to find different vectors for siRNA delivery [123]. At present, the diagnosis of liver fibrosis mainly depends on pathology and imaging examination. Although molecular markers of liver fibrosis are abundant [124], there are very few aptamers related to liver fibrosis. This is mainly because the progress of liver fibrosis is affected by many factors comprehensive, so it is used for the screening of aptamers of liver fibrosis and the application may be limited by a lot of restrictions, in Table 2, we only found two relevant aptamers, which is primarily on liver fibrosis in the process of increased protein expression on HSCs, and the treatment of siRNA has yet to achieve good results. The aptamer 20 obtained by insulin-like growth factor II receptor (IGFIIR) targeting performed well at the cellular level after carrying PCBP2 siRNA. Although IGFIIR is a non-specific marker of HSCs, it is overexpressed in activated HSCs and therefore was selected as the target. Aptamer-20 carrying siRNA into HSC-T6 can trigger the silencing effect and restore the activated HSC-T6 to the quiescent state [111]. For the screening of HSCs aptamer, in addition to HSC-T6, human LX-2 cell line or primary stellate cells isolated directly from mice can also be selected as positive screening cells, and better results will be obtained.

### Aptamers are Used in Diagnosis and Therapeutic of Liver Injury

Liver injury mainly includes trauma, acute injury caused by drugs, and chronic injury caused by viral infection. The main treatment for liver injury is liver transplantation, or the prevention and treatment of complications such as liver failure, without other specific treatment [125]. In the case of acute injury, hepatocyte necrosis leads to the secretion of TNF and the elevation of acute CRP. During liver failure, aptamer targets TNF and CRP mainly by inhibiting TNF and tracking the site of CRP secretion, thereby blocking the inflammatory process and reducing and eliminating inflammation [109, 126]. However, neither of these two targets is specific and can be increased by stress in other inflammatory states. Acute liver failure is rare, so it may still be misdiagnosed in diagnosis and treatment. Chronic liver injury is mainly caused by hepatitis B virus (HBV), hepatitis C virus (HCV), influenza, and other viruses [127]. SARS-CoV-2 can also affect the liver, but the main reason may be drug-induced liver injury caused by the use of antiviral drugs such as lopinavir/ritonavir, rather than the virus itself, and the exact mechanism has not been proved [128]. At present, for HBV and other infections, long-term nucleotide surimi, which is well tolerated and has few side effects is used, and the main preventive measure is vaccination without infection [129]. New treatments are still being developed, and aptamers are good candidates to carry antiviral drugs that can effectively treat HBV.

### Aptamers for Other Liver-Related Diseases

Many diseases can cause indirect damage to the liver in other ways. For example, cancer cells can migrate to the liver through the blood circulation and cause liver damage. Liver metastasis of cancer cells is more common than primary liver tumors, and liver metabolism is very vigorous. In the early stage of the disease, there are often no symptoms, and it is easy to miss the best treatment time [130]. At present, the main aim is to prevent the disease and reduce the involvement of the liver. Metron factor-1 (MF-1) has great potential to prevent some malignant diseases that are difficult to treat, including liver metastases, melanoma, gastrointestinal tumors, etc., because it inhibits angiogenesis and tumor metastasis [131].

Cells in the liver, including sinusoidal endothelial cells, HSCs, and kupff cells, communicate with cancer cells through complex cytokines that are potential therapeutic targets [132]. Schistosomiasis is a global health disease, and Schistosome infection can lead to complications such as liver fibrosis and portal hypertension [133]. At present, the aptamer LC15 obtained from Schistosoma japonicum egg screening can be used as a specific tool for accurate diagnosis and targeted therapy. It can carry specific drugs to kill Schistosoma japonicum eggs and effectively improve the serious health problems caused by Schistosoma parasite infection [134].

### Simulation of Aptamers and Targets

Aptamers enter cells by binding to protein targets on the cell surface and being internalized. Many targets have been identified, but how the target and aptamer interact is still not very clear, because the protein is difficult to isolate and purify, and even if isolated, it is difficult to maintain its original state [135]. Therefore, at present, the interaction of aptamers with their targets can only be inferred by calculating molecular simulation docking. This is generally done by constructing the secondary and tertiary structure of the aptamer, performing homology modeling of the corresponding target protein, and performing simulation docking on the Discovery Studio software to find possible modes of action according to the ranking of ZRANK scores [136]. Due to the secondary and tertiary structures of the aptamer fold, protein homology modeling differs from theory and is entirely computer simulated. Its authenticity has yet to be confirmed, but it can be used as a theoretical reference.

#### Secondary and Tertiary Structure Construction of Aptamer

Mfold web server (http://unafold.rna.albany.edu/?q=mfold) was used to predict and analyze the linear ssDNA aptamer secondary structure [137]. The optimal operating parameters are as follows: the folding temperature was controlled at 37℃, the ion conditions were Na^+^ 1.0 mM, Mg^2+^ 0.0 mM, the second-best percentage was 50%, the window parameters were default, and the maximum distance between paired bases was unrestricted by default. The aptamer structure with the smallest free energy, the smallest G value, was obtained. Select secondary structure Vienna format as a template to build the tertiary structure, in RNAcomposer (http://rnacomposer.ibch.poznan.pl/) generated in the tertiary structure, download to generate the tertiary structure of PDB format [138]. The nucleic acid sequence of the RNA tertiary structure was mutated using the biological software Discovery Studio to convert RNA into DNA sequence, and the structure was optimized [136, 137].

#### Target Protein Homology Modeling

In order to obtain information of each species-related genes in NCBI (https://www.ncbi.nlm.nih.gov/gene/) and to find the corresponding protein gene, the aptamer corresponding target protein was found. We chose homo sapiens (human) to find the amino acid sequence of the desired protein by the relationship between the corresponding gene and the protein. Amino acid sequence imports SWISS—MODEL (https://swissmodel.expasy.org/interactive) to carry on the homologous modeling, which can be directly chosen to establish a MODEL to get the corresponding protein template. To find out the optimal template search sequence, the most extensive coverage (generally more than 30%) and high matching degree should be used as a template. In Table 3, the GMQE, QMEAN, Seq identity, MolProbity Score, Ramachandran, Ramachandran Outliers, and solvation series parameters of the corresponding template are given, which are searched in the template to remove GP73 low credibility. Other validations were performed with high confidence, and the best homology template in PDB format was downloaded as the docking receptor.

**Table 3:** Protein parameters modeled

Name	Species	PDB ID	GMQE	QMEAN	Seq identity (%)	MolProbity score	Ramachandran favored (%)	Ramachandran outliers (%)	Solvation
GP73	Human	2d3e.1.A	0.13	− 1.02	21.15	1.52	95.73	0.00	1.72
AFB1	Human	2bp1.1.A	0.95	− 0.54	100.00	1.38	97.03	0.35	− 0.61
AFP	Human	4bke.1.A	0.75	− 1.09	40.13	1.38	97.41	0.17	2.31
GPC3	Human	4ywt.2.A	0.51	− 4.13	23.69	1.73	90.06	2.54	2.08
CRP	Human	1gnh.1.A	0.98	0.32	100.00	1.92	95.69	0.78	0.07
TNF-α	Human	2e7a.1.A	0.63	− 0.46	92.36	1.63	95.27	0.45	− 0.69
Nucleolin	*Cyprinus carpio*	2krr.1.A	0.34	− 2.01	54.88	2.85	90.42	0.60	− 0.02
hnRNP A1	Human	2lyv.1.A	0.69	− 1.58	100.00	1.33	98.95	0.00	− 1.07
EGFR	Human	3qwq.1.A	0.59	0.14	99.83	1.78	94.35	0.53	0.05
eEF1A1	Human	5lzs.78.A	0.97	− 0.96	100.00	1.40	96.35	0.23	0
CD105	Human	5hzw.1.A	0.48	− 0.49	100.00	0.82	96.37	0.33	− 0.65
LDL-R	Human	3p5c.1.C	0.51	− 3.48	100.00	2.52	83.91	2.76	− 2.02

*GP73* Golgi protein 73, *AFB1* Aflatoxin B1, *AFP* Alpha-fetoprotein, *GPC3* Glypican 3, *TNF-α* Tumor Necrosis Factor α, *CRP* C-reactive protein, *hnRNP A1* Heterogeneous nuclear ribonucleoproteins, *EGFR* Epidermal growth factor receptor, *eEF1A1* Eukaryotic elongation factor 1A1, *LDL-R* Low-Density Lipoprotein-Cholesterol

#### Molecular Simulation Docking Between Ligand Receptors

**Fig. 2:**
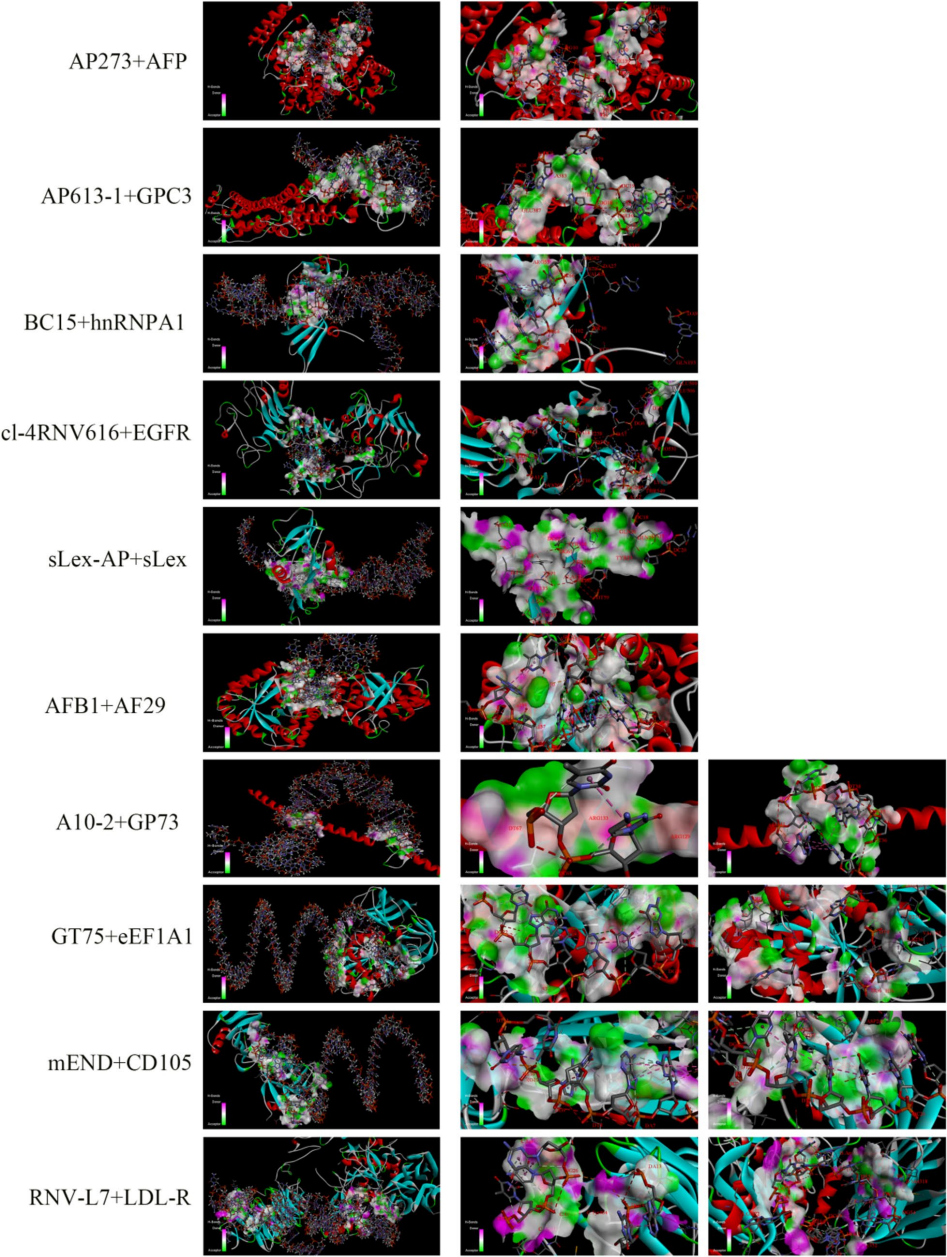
Protein–aptamer docking diagram. AP273 + AFP; AP613-1 + GPC3; BC15 + hnRNPA1; cl-4RNV616 + EGFR; sLEX-AP + sLEX; AFB1 + AF29; A10-2 + GP73; GT75 + eEF1A1; mEND + CD105; RNV-L7 + LDL-R

Docking of nucleic acid aptamers and proteins was performed in Discovery Studio. The water molecules on the surface of the protein were first removed and re-hydrogenated and structurally optimized in chemistry as acceptors. Before docking with Dock Proteins (ZDOCK), you need to choose enough parameters, including selecting “Angular Step size” to be more extensive and detailed 6 instead of 15, selecting “Zrank” to be true, and selecting “Angular step size” to be more extensive and detailed 6 instead of 15. Select “Parallel Processing” to false, and ZDOCK runs. After completion of docking, the best docking result was selected according to the ZRANK scoring order [136]. By changing different docking methods, ZRANK scores of corresponding structural maps were obtained, and the interaction modes of aptamers and proteins were analyzed. Whether the motif of the aptamer binds to the target protein in a similar way to that of the contrast antibody is not known, nor is it clear that it is consistent with the results of computer simulations. In general, lower ZRANK scores indicate stronger receptor–ligand interactions [139]. The parameters and images corresponding to the docking results are shown in Table 4 and Fig. 2, respectively.

**Table 4:** Results parameters of protein–aptamer docking

Name	Target	Docking site	Sequence	ZRank score
A10-2	GP73	ASP101-DG34 LEU98-DT36,DA37 LYS103-DA37,DT38 VAL107-DT39 LEU106-DT40 THR112-DT40 ARG115-DA41, DT40ARG133-DT67 GLN136-DT67,DC68 ARG129-DC68 GLY132-DC68	ACGCTCGGATG CCACTACAGTT GGTTTTTTTTTG TTATTTAGAGTA AAAACCTTGTG TGTAGTGACTC ATGGACGTGCT GGTGACACCTT GTGTGTAGTGA CTCATGGACGTGCTGGTGAC	− 86.96
AF29	AFB1	THY174-DT1 PRO178-DT1 PHE323-DT1 GLU175-DT1,DG2 HIS145-DG2 TYR322-DG2 ARG324-DG2 GLN161-DC3 PHE183-DA4 LEU185-DA4 HIS160-DC5 LEU153-DG6 GLN157-DT7 HIS154-DT9,DT7,DG8 PRO148-DT24 GLU151-DT24 GLU150-DG8,DT24,DT7,DC25 CYS179-DG28,DC3,DC29 HIS145-DC29 Protein A chain Protein B chain	TGCACGTGTTG TCTCTCTGTGT CTCGTGC	− 117.515
AP273	AFP	PRO131-DT6 THR132-DT6 GLN221-DT6 PHE172-DG7 SER217-DG7 SER217-DG7 PRO133-DT6,DG7 HIS483-DC8 ARG214-DA9 ALA449-DA9 ARG452-DG10 THR547-DG10 SER445-DT19 SER444-DC20 LYS558-DC20 LYS530-DG21 ASN555-DG21 ASP529-DG22 MET548-DG22 LEU138-DG27 PHE139-DG27,DA28 DLN140-DG27,DA28 PRO142-DA28 GLU143-DG30 THR146-DG30 GLU149-DT31	TCAGGTGCAGT TCTCGACTCGG TCTTGATGTGG GT	− 95.349
AP613-1	GPC3	ARG381-DC6 SER382-DT7,DC6 ALA383-DT7,DC6 PRO386-DA14,DT7 GLU387-DG8,DA14 ASN184-DT17 PRO185-DT17 GLN379-DG18 GLU345-DC19,DG18 PHE359-DC19 THR346-DA20,DC19 GLU347-DC19,DA20 LYS349-DA20,DT21 TRY380-DT27 ILE357-DG32,DT33,DC19 PRO356-DT34 PHE358-DT34	TAACGCTGACC TTAGCTGCATG GCTTTACATGT TCCA	− 116.122
AS1411	Nucleolin	GLU194-DG10 ASP176-DG10,DG16 GLN164-DG16 ILE177-DG16 ARG178-DG16,DG17,DT13 ARG178-DG16,DG17,DT13 MET181-DG17 LEU132-DG19 ARG188-DG19 ASP133-DG20,DG19 LEU118-DG20 GLN115-DT21 LYS226-DT21 SER227-DG22,DG23,DT21 GLU147-DG25 ARG148-DG25 SER144-DG26	GGTGGTGGTGG TTGTGGTGGTG GTGG	− 77.687
BC15	hnRNP A1	GLN193-DA9 LYS78-DA27 VAL83-DA27 ARG82-DC66,DA27 SER188-DT30 ARG92-DG38 GLN96-DG38,DT39,DG62 SER95-DC63 ARG97-DC63 PRO98-DC63 ALA100-DC63 HIS101-DC63,DT64 LEU102-DT64 PHE57-DA65 GLY20-DC66 ARG55-DG35,DA65,DG36,DC66	GCAATGGTACG GTACTTCCTGT GGCGAGGTAG GTGGGGTGTG TGTGTATCCAA AAGTGCACGCT ACTTTGCTAA	− 86.841
cl-4RNV616	EGFR	ASN507-DT3 GLU509-DT3 GLU506-DT3,DT4 ARG529-DT5,DT4 LYS282-DG6 MET232-DA7 ASP258-DT8 LYS208-DT10 TYR112-DT14,DA13 LYS37-DT14 TYR88-DT14 MET111-DT14,DT15 GLN32-DT15 SER35-DT15 ARG264-DC16,DT15 ASP269-DG17,DA18 LYS548-DA23,DG21 PRO531-DG22 VAL554-DG24,DA23 THR549-DG24 PRO551-DG24 ALA552-DG24	GmCmUmUmUm GdAmUmGmUm CmGdAmUmUm CmGdAmCdAm GmGdAmGmGmC	− 97.804
GT75	eEF1A1	LEU63-DG2 ASP74-DG2 ARG322-DG2 TYR254-DT4 ILE256-DT4,DT5 VAL262-DT5 HIS296-DT5,DG6 LYS41-DT8 LYS44-DT8 GLU45-DT8 ASP97-DG18 VAL16-DT21 GLY121-DT21 ALA125-DT21 ASP17-DT21,DG22 GLU122-DT21,DG22 SER157-DT23 SER53-DT24 ASP156-DT24 GLY50-DT25 GLY52-DT25 ALA47-DG26 GLU48-DG26	TGTTTGTTTGT TTGTTTGTTTG TTTGTTTGTTT GTTTGTTTGTT TGTTTGTTTGT TTGTTTGTTTG TTTGTTTGT	− 97.537
mEND	CD105	SER309-DC2 HIS208-DC3 ARG39-DG6,DC5 GLU38-DA7,DG6 PRO37-DA7,DT8 GLU41-DT8 VAL42-DG9 PRO201-DG9 GLN77-DC10 ARG199-DC10,DT11 GLU79-DT11 GLY214-DC22,DT23 HIS215-DT23,DC22 ASP248-DT24,DT25 ASP246-DC26 ASN268-DC26 ASN266-DG27 HIS267-DG27	CCCCCGATGCT TTCGCTTTTCC TTTCGCTTTTG TTCGCTTCGTC CCTGGTTCCTT TCTTG	− 117.225
RNV-L7	LDL-R	A: GLU72-DA45 PHE4-DC47,DG48 THR3-DG48 HIS5-DA49,DG48 ALA8-DA49 THR1-DC65,DT64 B: TYR141-DG50,DA49 ARG205-DG51 HIS312-DG51 SER313-DG51,DT52 ALA319-DT52 ALA321-DT52 THR320-DT52,DA53 MET318-DA53 ARG358-DA53,DG54 GLY352-DG61 ARG324-DG62,DC63 ARG143-DT64 C: VAL231-DA13 TYR404-DC14 ARG434-DT24 LEU397-DC25,DT24 GLY401-DT24,DC25 CYS402-DC25 GLN403-DC25 SER398-DC25,DG26 LYS232-DA13,DC14,DG26	GGACAGGACCA CACCCAGCGCG GTCGGCGGGTG GGCGGGGGGA GAACGAGGTAG GGGTCAGGCTC CTGTGTGTCGC TTTGT	− 102.592
sLeX-AP	sLeX	ASN31-DT8 ILE137-DT8 TRP1-DG9,DT8 GLU132-DA10 CYS133-DA10 VAL134-DA10 GLN20-DC18 GLN21-DC20,DA19 TYR23-DA19,DT59 HIS25-DG60 ALA120-DG60 THR119-DT59,DG60 GLU135-DA10,DG60 TYR118-DG61 THR65-DC62 LYS67-DA63,DG64	ATGACCATGAC CCTCCACACGT TTTTGTGTGCA TGTGACGCTTG TATGATTCAGA CTGTGGCAGG GAAAC	− 98.866

*GP73* Golgi protein 73, *AFB1* Aflatoxin B1, *AFP* Alpha-fetoprotein, *GPC3* Glypican 3, *hnRNP A1* Heterogeneous nuclear ribonucleoproteins, *EGFR* Epidermal growth factor receptor, *eEF1A1* Eukaryotic elongation factor 1A1, *LDL-R* Low-Density Lipoprotein-Cholesterol, *sLeX* Sialyl Lewis X

## Discussion

Aptamers bind cells in an antibody-like manner, recognize cell-specific targets, enter the cell by endocytosis to form vesicles, and after entering the cell, some of the aptamers escape from the vesicles. Some aptamers function in the cytoplasm, while others enter the nucleus [140]. Since their discovery, aptamers have attracted much attention due to their non-toxicity, low immunogenicity, easy penetration of tissue barriers, and the advantages of multiple targets (amino acids, peptides, proteins [4, 10, 14], antibiotics [15], cells, viruses [20, 23], bacteria [24, 26]). At present, the defects of aptamer and SELEX technology hinder the utilization degree of aptamer. Improving the screening process and optimizing aptamer may make new breakthroughs in aptamer research [141]. Despite many setbacks in research, it has been used as a potential alternative to antibodies and diagnostic reagents in biomedical applications such as disease diagnosis, molecular imaging, drug delivery, biomarker discovery, and drug screening [142]. By summarizing the aptamers that have been patented so far, we get a general idea of the current state of research. Although many aptamers have been discovered, they are mostly used in other industries. In medicine, it is still in the stage of basic research, and there is still a gap between it and clinical diagnosis and treatment. In the process of screening aptamers, the biological characteristics such as specificity, affinity, stability, truncation and modification, carrying decoy ODN, micro-RNA or siRNA, etc., still need to be determined, which increases the cost of research and leads to the bottleneck of research. At present, the emergence of a variety of bioanalysis software provides convenience and support for related research. In addition to comparing with the existing experimental results, it can also provide certain guidance for the experiment. 

## Conclusion

The high affinity, targeting specificity, and cell internalization ability exhibited by aptamers are key to their application in drug delivery and the treatment of liver diseases. They can search for different target proteins and be applied to the diagnosis and treatment of liver diseases through screening corresponding aptamers.

### Supplementary Information

Below is the link to the electronic supplementary material.

Supplementary file1 (DOCX 267 KB)

## Data Availability

The datasets analyzed during this study are available from the corresponding author on reasonable request.
