# Supplementary material for: The Application of Aptamer and Research Progress in Liver Disease
Source: Mol Biotechnol. 2024 Feb 2;66(5):1000–18. doi: 10.1007/s12033-023-01030-4 (PMC11087326; doi:10.1007/s12033-023-01030-4)
Supplement: Supplementary file 1 — Supplementary file1 (DOCX 267 KB) [file 12033_2023_1030_MOESM1_ESM.docx]

**supplementary material**
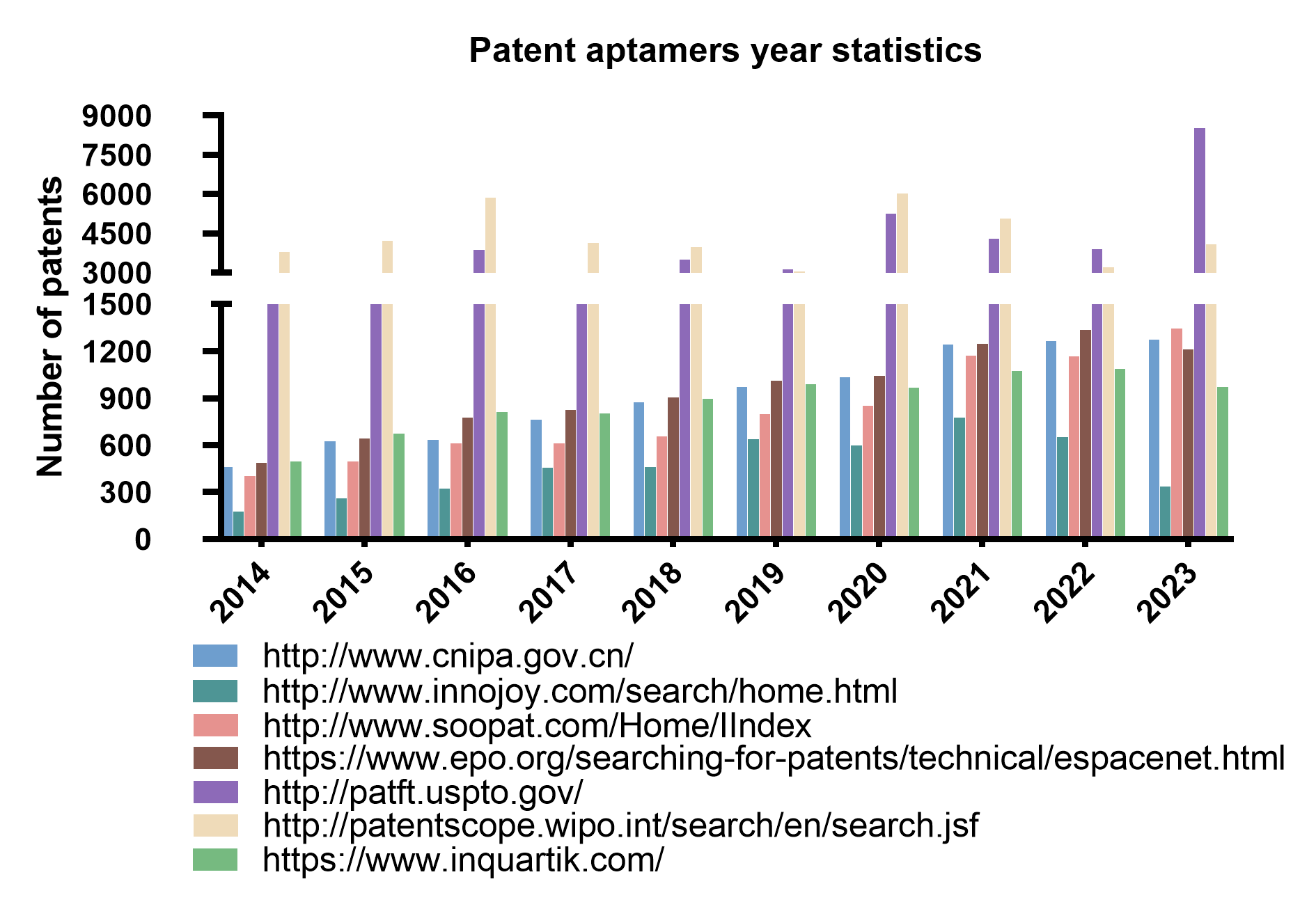


**Fig. S1** The numbers of patent since 2014. Keyword included: "aptamers".

**Table S1** markers of liver diseases

| **diseases** | **biomarker** | **location** | **description** | **ref** |
| --- | --- | --- | --- | --- |
| liver  cancer | AFP | serum | major serum markers of primary liver cancer | ^［1］^ |
|  | AFP-L3 | serum | AFP binding fraction, derived only from cancer cells, are more specific markers | ^［2］^ |
|  | DCP | serum | prothrombin molecule that may be more sensitive than AFP | ^［3］^ |
|  | ctDNA | blood | tumors can be detected using ctDNA in the blood | ^［4］^ |
|  | Dickkopf-1 | membrane | the antagonist of Wnt/b-catenin signaling pathway can combine with AFP to improve the detection rate of HCC | ^［5］^ |
|  | GP73 | membrane | potential biomarkers for early diagnosis are more sensitive than AFP | ^［6］^ |
|  | GPC3 | membrane | HCC is up-regulated and can be used as an immune-specific target | ^［7］^ |
|  | long non- coding RNA p34822 | plasma | diagnosis of potential markers of HCC | ^［8］^ |
|  | MDK | serum | cell growth, survival, migration, angiogenesis and cancer play key roles | ^［9］^ |
|  | micro-RNA | plasma | many multi-functional micro-RNA can be used as early diagnosis, prognostic indicators and therapeutic targets for HCC | ^［10］^ |
|  | OPN | membrane | HCC is up-regulated and can be used as a diagnostic marker for early HCC | ^［11］^ |
|  | SCCA | tissues/ body/fluids/excreta | the potential markers of liver cancer, high sensitivity and low specificity can be used as a supplement of AFP | ^［12］^ |
|  | vesicles | extracellular | as a candidate for liquid biopsy of tumor | ^［13］^ |
|  | Annexin A2 | serum | combination with AFP can improve sensitivity and specificity, and can be used as an independent serum candidate markers for early HCC | ^［14］^ |
|  | suPAR | serum | biomarkers for cancer metastasis, potentially evaluating early indicators of HCC | ^［14］^ |
|  | AXL | serum | the sensitivity was higher than that of AFP, which was used to detect early HCC | ^［15］^ |
|  | CXCR2, CCR2, EP400 | serum | three genes are combined with AFP to predict HCC, which is more efficient than AFP detection | ^［16］^ |
|  | CD133 | membrane | membrane glycoproteins, markers of cancer stem cells (CSC) | ^［17］^ |
|  | ALDH | intracellular | similar to CD133 expression, CSC can be better detected with CD133 | ^［18］^ |
|  | CD90 | membrane | potential markers for CSC | ^［19］^ |
|  | CD44 | membrane | CD44 can be used with other markers to detect liver CSCS | ^［20］^ |
|  | CD13 | membrane | CD13 is a marker of liver cancer cells, and CD13 inhibitors can relieve liver cancer | ^［21］^ |
|  | EpCAM | membrane | early markers of liver cancer are related to the prognosis of liver cancer | ^［22］^ |
|  | OV-6 | membrane | liver stem cell markers, Wnt pathway intervention, may be the treatment of ov-6 positive tumors | ^［23］^ |
|  | 1B50-1 | membrane | 1B50-1 cells are associated with tumorigenesis | ^［24］^ |
|  | SALL4 | membrane | hepatocyte marker is a new therapeutic target for hepatocellular carcinoma | ^［25］^ |
|  | ICAM-1 | membrane | ICAM-1 is a marker of HCC hepatocytes, and inhibition of ICAM-1 can delay tumor development | ^［26］^ |
| liver  failure | miR-223-3p and miR-25-3p | blood | miR-223-3p and miR-25-3p are associated with survival, prognosis and mortality in the process of chronic liver failure | ^［27］^ |
|  | caspase-1 | serum | it is a biomarker for the detection of ACLF progression and prognosis | ^［28］^ |
|  | Plasminogen | serum | prognostic markers of HBV-ACLF | ^［29］^ |
|  | FABP1 | serum | as a potential marker for acute liver failure | ^［30］^ |
|  | NGAL | plasma | ACLF is a prognostic biomarker associated with liver failure and inflammation | ^［31］^ |
|  | CSF1 | serum | as a new prognostic marker of ALF, it has therapeutic potential | ^［32］^ |
|  | M30- and M65- antigens | serum | prognostic index of HBV ACLF | ^［33］^ |
|  | AFP | serum | ALF serum markers | ^［34］^ |
|  | glycodeoxycholic acid | serum | prognostic markers of ALF | ^［35］^ |
|  | HDL-C and apoA-1 | serum | predictors of chronic liver failure | ^［36］^ |
|  | leukocyte telomere length | chromatin | as a potential marker for early ATDILI | ^［37］^ |
|  | MCP-1 | serum | MCP-1 mainly comes from the damaged liver and is related to the degree of damage | ^［38］^ |
|  | GLDH | serum | sensitive markers of liver injury related to mitochondrial function | ^［39］^ |
|  | kallistatin | serum | a reliable diagnostic indicator of liver health status | ^［40］^ |
|  | CK18/CCK18 | intracellular/serum | the ratio of CK18 to ccK18 reflects hepatocyte necrosis and apoptosis | ^［41］^ |
|  | GSTα | cytoplasm | GST isoenzyme, associated with ACLF prognosis | ^［42］^ |
|  | BAs | bile canaliculi | DILI markers, but limited specificity | ^［43］^ |
|  | CYP450 | endoplasmic reticulum | the expression of CYP450 and isoenzyme is related to liver injury | ^［44］^ |
|  | OPN | extracellular | associated with ALF and associated with prognosis | ^［45］^ |
|  | HMGB1 | nucleus | in most tissues, acetylated HMGB1 may be a prognostic indicator of DILI | ^［46］^ |
|  | FABP1 | cytoplasm | low expression of FABP1 prevents liver cell damage | ^［30］^ |
|  | CDH5 | cell membrane | the concentration increases with liver injury | ^［47］^ |
|  | miR-122 | cytoplasm | the expression was increased during liver injury, but the specificity for early liver injury was low | ^［48］^ |
|  | ITGB3 | blood | specific biomarkers for drug-induced liver injury | ^［49］^ |
| liver fibrosis | MFAP4 | blood | MFAP4 can detect liver fibrosis caused by alcohol injury | ^［50］^ |
|  | hyaluronic acid/type IV collagen | blood | assess the degree of liver fibrosis | ^［51］^ |
|  | APRI | blood | prediction of liver fibrosis | ^［52］^ |
|  | FIB-4 | blood | a simple combination marker for HCV infection | ^［52］^ |
|  | fibroIndex | blood | the prediction of fibrosis severity can be used as an alternative index of anti-fibrosis | ^［53］^ |
|  | ELF | blood | OELF improvement, fibrosis evaluation | ^［54］^ |
|  | fibro test | blood | quantitative estimation of fibrosis predicts late fibrosis | ^［55］^ |
|  | BAAT score | blood | discrete biomarkers for liver fibrosis | ^［56］^ |
|  | NAFLD fibrosis score | blood | noninvasive prediction of hepatic fibrosis | ^［57］^ |
|  | fibrometer NAFLD | blood | noninvasive assessment of accurate detection of liver fibrosis | ^［57］^ |
|  | NAFIC score | serum | diagnosis of nonalcoholic steatohepatitis | ^［58］^ |
|  | FPS | blood | hepatitis severity and advanced fibrosis were predicted | ^［59］^ |
|  | miR-185 | serum | markers of early liver fibrosis | ^［60］^ |
|  | visceral adiposity | cytoplasm | clinical predictors of fibrosis | ^［61］^ |
| HBV | HBsAg | serum | immune cell biomarkers in patients with chronic hepatitis B | ^［62］^ |
|  | plasminogen | plasma | prognostic markers of acute onset of HBV | ^［63］^ |
|  | D-dimer | plasma | prognostic marker for compensation of cirrhosis caused by HBV | ^［64］^ |
|  | caspase-1 | serum | HBV causes different expression in related liver diseases | ^［28］^ |
|  | β-catenin | serum | HBV infection enhances the expression of N-catenin and may be used to distinguish between stages of HBV disease | ^［65］^ |
|  | NOX2 | serum | the positive correlation with HBV virus quantity may be an indicator of the pathogenesis of HBV disease | ^［66］^ |
|  | GP73 | cell membrane | chronic HBV infection causes liver fibrosis biomarkers | ^［67］^ |
|  | miR-185 | serum | HBV causes early liver fibrosis markers | ^［60］^ |
| HCV | HCV immunoglobulin G antibody | plasma | estimated incidence of HCV infection | ^［68］^ |
|  | miR-122 | serum | miR-122 is a replication regulator of HCV and can be used as an antiviral marker | ^［69］^ |
|  | complement C3a | serum | as a biomarker candidate for chronic hepatitis C | ^［70］^ |

**Note:** AFP: Alpha-fetoprotein; DCP: Des-γ-carboxy prothrombin; GP73: Golgi protein 73; GPC3: Glypican 3; MDK: Midkine; OPN: Osteopontin; SCCA: Squamous cell carcinoma antigen; suPAR: Soluble urokinase plasminogen activator receptor; CXCR2: C-X-C motif chemokine receptor 2; EP400: E1A binding protein p400; CD133: Prominin-1; ALDH: Aldehyde dehydrogenase; EpCAM: Epithelial Cell Adhesion Molecule; SALL4: Spalt-Like Transcription Factor 4; ICAM-1: Intercellular adhesion molecule-1; FABP1: Fatty acid-binding protein 1; NGAL: Neutrophil Gelatinase Associated Lipocalin; CSF1: colony-stimulating factor 1; MCP-1: Monocyte chemoattractant protein-1; GLDH: Glutamate dehydrogenase; CK18: Cytokeratin 18; ccK18: Caspase-cleaved keratin 18; BAs: Bile acids; OPN: Osteopontin; HMGB1: High mobility group box-1 protein; CDH5: Cadherin 5; ITGB3 β3 integrin; MFAP4: Microfibrillar-associated protein 4; APRI: Aspartate aminotransferase/platelet ratio index; FIB-4: Fibrosis-4; ELF: Enhanced liver fibrosis; BAAT: BMI, age, alanine aminotransferase, and triglycerides; NAFLD: Nonalcoholic fatty liver disease; NOX2: NADPH oxidases 2.

**Reference**

1. Trevisani, F., Garuti, F., & Neri, A. (2019). Alpha-fetoprotein for Diagnosis, Prognosis, and Transplant Selection. *Seminars in liver disease, 39*(2):163-177.
2. Park, S.J., Jang, J.Y., Jeong, S.W., Cho, Y.K., Lee, S.H., Kim, S.G., Cha, S.W., Kim, Y.S., Cho, Y.D., Kim, H.S., Kim, B.S., Park, S., & Bang, H.I. (2017). Usefulness of AFP, AFP-L3, and PIVKA-II, and their combinations in diagnosing hepatocellular carcinoma. *Medicine, 96*(11):e5811.
3. Wong, R.J., Ahmed, A., & Gish, R.G. (2015). Elevated alpha-fetoprotein: differential diagnosis-hepatocellular carcinoma and other disorders. *Clinics in liver disease, 19*(2):309-323.
4. Xu, R.H., Wei, W., Krawczyk, M., Wang, W., Luo, H., Flagg, K., Yi, S., Shi, W., Quan, Q., Li, K., Zheng, L., Zhang, H., Caughey, B.A., Zhao, Q., Hou, J., Zhang, R., Xu, Y., Cai, H., Li, G., Hou, R., Zhong, Z., Lin, D., Fu, X., Zhu, J., Duan, Y., Yu, M., Ying, B., Zhang, W., Wang, J., Zhang, E., Zhang, C., Li, O., Guo, R., Carter, H., Zhu, J.K., Hao, X., & Zhang, K. (2017). Circulating tumour DNA methylation markers for diagnosis and prognosis of hepatocellular carcinoma. *Nat Mater. 16*(11):1155-1161.
5. Jeng, J.E., Chuang, L.Y., Chuang, W.L., & Tsai, J.F. (2012). Serum Dickkopf-1 as a biomarker for the diagnosis of hepatocellular carcinoma. *Chinese clinical oncology, 1*(1):4.
6. Zhang, Y., Xi, Y., Fang, J., Luo, S., Wilson, J.J., & Huang, R.P. (2016). Identification and characterization of monoclonal antibodies against GP73 for use as a potential biomarker in liver cancer screening and diagnosis. *Journal of immunoassay & immunochemistry, 37*(4):390-406.
7. Zhou, F., Shang, W., Yu, X., & Tian, J. (2018). Glypican-3: A promising biomarker for hepatocellular carcinoma diagnosis and treatment. *Medicinal research reviews, 38*(2):741-767.
8. Wang, C., Ren, T., Wang, K., Zhang, S., Liu, S., Chen, H., & Yang, P. (2017). Identification of long non-coding RNA p34822 as a potential plasma biomarker for the diagnosis of hepatocellular carcinoma. *Sci China Life Sci. 60*(9):1047-1050.
9. Zhu, W.W., Guo, J.J., Guo, L., Jia, H.L., Zhu, M., Zhang, J.B., Loffredo, C.A., Forgues, M., Huang, H., Xing, X.J., Ren, N., Dong, Q.Z., Zhou, H.J., Ren, Z.G., Zhao, N.Q., Wang, X.W., Tang, Z.Y., Qin, L.X., & Ye, Q.H. (2013). Evaluation of midkine as a diagnostic serum biomarker in hepatocellular carcinoma. *Clin Cancer Res. 19*(14):3944-54.
10. Thakral, S., & Ghoshal, K. (2015). miR-122 is a unique molecule with great potential in diagnosis, prognosis of liver disease, and therapy both as miRNA mimic and antimir. *Current gene therapy, 15*(2):142-150.
11. Cao, D.X., Li, Z.J., Jiang, X.O., Lum, Y.L., Khin, E., Lee, N.P., Wu, G.H., & Luk, J.M. (2012). Osteopontin as potential biomarker and therapeutic target in gastric and liver cancers. *World J Gastroenterol. 18*(30):3923-30.
12. Liu, C.H., Gil-Gómez, A., Ampuero, J., & Romero-Gómez, M. (2018). Diagnostic accuracy of SCCA and SCCA-IgM for hepatocellular carcinoma: A meta-analysis. *Liver international : official journal of the International Association for the Study of the Liver, 38*(10):1820-1831.
13. Liu, H., & Li, B. (2018). The functional role of exosome in hepatocellular carcinoma. *Journal of cancer research and clinical oncology, 144*(11):2085-2095.
14. El-Abd, N., Fawzy, A., Elbaz, T., & Hamdy, S. (2016). Evaluation of annexin A2 and as potential biomarkers for hepatocellular carcinoma. *Tumour biology : the journal of the International Society for Oncodevelopmental Biology and Medicine, 37*(1):211-216.
15. Staufer, K., Dengler, M., Huber, H., Marculescu, R., Stauber, R., Lackner, C., Dienes, H.P., Kivaranovic, D., Schachner, C., Zeitlinger, M., Wulkersdorfer, B., Rauch, P., Prager, G., Trauner, M., & Mikulits, W. (2017). The non-invasive serum biomarker soluble Axl accurately detects advanced liver fibrosis and cirrhosis. *Cell death & disease, 8*(10):e3135.
16. Shi, M., Chen, M.S., Sekar, K., Tan, C.K., Ooi, L.L., & Hui, K.M. (2014). A blood-based three-gene signature for the non-invasive detection of early human hepatocellular carcinoma. *European journal of cancer (Oxford, England : 1990), 50*(5):928-936.
17. Jun, S.Y., Jeon, S.J., Yoon, J.Y., Lee, J.J., Yoon, H.R., Choi, M.H., Halder, D., Lee, K., & Kim, N.S. (2019). The positive correlation of TIPRL with LC3 and CD133 contributes to cancer aggressiveness: potential biomarkers for early liver cancer. *Scientific reports, 9*(1):16802.
18. Chen, X., Lingala, S., Khoobyari, S., Nolta, J., Zern, M.A., & Wu, J. (2011). Epithelial mesenchymal transition and hedgehog signaling activation are associated with chemoresistance and invasion of hepatoma subpopulations. *Journal of hepatology, 55*(4):838-845.
19. Zhang, K., Che, S., Su, Z., Zheng, S., Zhang, H., Yang, S., Li, W., & Liu, J. (2018). CD90 promotes cell migration, viability and sphere‑forming ability of hepatocellular carcinoma cells. *International journal of molecular medicine, 41*(2):946-954.
20. Mustika, S., Wijaya, H., & Pratomo, B. (2019). The Expressions of CD44, CD90 and Alpha Fetoprotein Biomarkers in Indonesian Patients with Advanced Liver Disease: an Observational Study. *Acta medica Indonesiana, 51*(2):137-144.
21. Yamanaka, C., Wada, H., Eguchi, H., Hatano, H., Gotoh, K., Noda, T., Yamada, D., Asaoka, T., Kawamoto, K., Nagano, H., Doki, Y., & Mori, M. (2018). Clinical significance of CD13 and epithelial mesenchymal transition (EMT) markers in hepatocellular carcinoma. *Japanese journal of clinical oncology, 48*(1):52-60.
22. Noh, C.K., Wang, H.J., Kim, C.M., Kim, J., Yoon, S.Y., Lee, G.H., Cho, H.J., Yang, M.J., Kim, S.S., Hwang, J.C., Cho, S.W., Roh, J., Kim, Y.B., Kim, S.J., Kim, B.W., & Cheong, J.Y. (2018). EpCAM as a Predictive Marker of Tumor Recurrence and Survival in Patients Who Underwent Surgical Resection for Hepatocellular Carcinoma. *Anticancer research, 38*(7):4101-4109.
23. Zhu, J., Yu, H., Chen, S., Yang, P., Dong, Z., Ling, Y., Tang, H., Bai, S., Yang, W., Tang, L., Shen, F., Wang, H., & Wen, W. (2018). Prognostic significance of combining high mobility group Box-1 and OV-6 expression in hepatocellular carcinoma. *Science China. Life sciences, 61*(8):912-923.
24. Zhao, W., Wang, L., Han, H., Jin, K., Lin, N., Guo, T., Chen, Y., Cheng, H., Lu, F., Fang, W., Wang, Y., Xing, B., & Zhang, Z. (2013). 1B50-1, a mAb raised against recurrent tumor cells, targets liver tumor-initiating cells by binding to the calcium channel α2δ1 subunit. *Cancer cell, 23*(4):541-556.
25. Yin, F., Han, X., Yao, S.K., Wang, X.L., & Yang, H.C. (2016). Importance of SALL4 in the development and prognosis of hepatocellular carcinoma. *World journal of gastroenterology, 22*(9):2837-2843.
26. Zhu, X., & Gong, J. (2013). Expression and role of icam-1 in the occurrence and development of hepatocellular carcinoma. *Asian Pacific journal of cancer prevention : APJCP, 14*(3):1579-1583.
27. Cisilotto, J., do Amaral, A.E., Rosolen, D., Rode, M.P., Silva, A.H., Winter, E., da Silva, T.E., Fischer, J., Matiollo, C., Rateke, E.C.M., Narciso-Schiavon, J.L., Schiavon, L.L., & Creczynski-Pasa, T.B. (2020). MicroRNA profiles in serum samples from Acute-On-Chronic Liver Failure patients and miR-25-3p as a potential biomarker for survival prediction. *Scientific reports, 10*(1):100.
28. Zhang, X., Dong, P., Xu, L., Tian, Y., Sun, H., Shi, H., Duan, Z., Chen, L., & Ren, F. (2019). The different expression of caspase-1 in HBV-related liver disease and acts as a biomarker for acute-on-chronic liver failure. *BMC gastroenterology, 19*(1):148.
29. Wu, D., Zhang, S., Xie, Z., Chen, E., Rao, Q., Liu, X., Huang, K., Yang, J., Xiao, L., Ji, F., Jiang, Z., Zhao, Y., Ouyang, X., Zhu, D., Dai, X., Hou, Z., Liu, B., Deng, B., Zhou, N., Gao, H., Sun, Z., & Li, L. (2020). Plasminogen as a prognostic biomarker for HBV-related acute-on-chronic liver failure. *J Clin Invest. 130*(4):2069-2080.
30. Fan, H., & Zhu, J. (2017). FABP1 as a novel potential biomarker for predicting mortality in acetaminophen-induced acute liver failure. *Hepatology. 66*(2):670
31. Ariza, X., Graupera, I., Coll, M., Solà, E., Barreto, R., García, E., Moreira, R., Elia, C., Morales-Ruiz, M., Llopis, M., Huelin, P., Solé, C., Fabrellas, N., Weiss, E., Nevens, F., Gerbes, A., Trebicka, J., Saliba, F., Fondevila, C., Hernández-Gea, V., Fernández, J., Bernardi, M., Arroyo, V., Jiménez, W., Deulofeu, C., Pavesi, M., Angeli, P., Jalan, R., Moreau, R., Sancho-Bru, P., Ginès, P.; CANONIC Investigators, & EASL CLIF Consortium. (2016). Neutrophil gelatinase-associated lipocalin is a biomarker of acute-on-chronic liver failure and prognosis in cirrhosis. *Journal of hepatology, 65*(1):57-65.
32. Tacke, F., & Wynn, T.A. (2015). Biomarker and Therapeutic Potential of CSF1 in Acute Liver Failure. *Gastroenterology. 149*(7):1675-8.
33. Cao, Z., Li, F., Xiang, X., Liu, K., Liu, Y., Tang, W., Lin, L., Guo, Q., Bao, S., Xie, Q., & Wang, H. (2015). Circulating cell death biomarker: good candidates of prognostic indicator for patients with hepatitis B virus related acute-on-chronic liver failure. *Scientific reports, 5*:14240.
34. Kakisaka, K., Kataoka, K., Onodera, M., Suzuki, A., Endo, K., Tatemichi, Y., Kuroda, H., Ishida, K., & Takikawa, Y. (2015). Alpha-fetoprotein: A biomarker for the recruitment of progenitor cells in the liver in patients with acute liver injury or failure. *Hepatology research : the official journal of the Japan Society of Hepatology, 45*(10):E12-E20.
35. Woolbright, B.L., McGill, M.R., Staggs, V.S., Winefield, R.D., Gholami, P., Olyaee, M., Sharpe, M.R., Curry, S.C., Lee, W.M., Jaeschke, H.; & Acute Liver Failure Study Group. (2014). Glycodeoxycholic acid levels as prognostic biomarker in acetaminophen-induced acute liver failure patients. *Toxicological sciences : an official journal of the Society of Toxicology, 142*(2):436-444.
36. Trieb, M., Rainer, F., Stadlbauer, V., Douschan, P., Horvath, A., Binder, L., Trakaki, A., Knuplez, E., Scharnagl, H., Stojakovic, T., Heinemann, Á., Mandorfer, M., Paternostro, R., Reiberger, T., Pitarch, C., Amorós, A., Gerbes, A., Caraceni, P., Alessandria, C., Moreau, R., Clària, J., Marsche, G., & Stauber, R.E. (2020). HDL-related biomarkers are robust predictors of survival in patients with chronic liver failure. *Journal of hepatology, 7*3(1):113-120.
37. Udomsinprasert, W., Chanhom, N., Suvichapanich, S., Wattanapokayakit, S., Mahasirimongkol, S., Chantratita, W., & Jittikoon, J. (2020). Leukocyte telomere length as a diagnostic biomarker for anti-tuberculosis drug-induced liver injury. *Scientific reports, 10*(1):5628.
38. Queck, A., Bode, H., Uschner, F.E., Brol, M.J., Graf, C., Schulz, M., Jansen, C., Praktiknjo, M., Schierwagen, R., Klein, S., Trautwein, C., Wasmuth, H.E., Berres, M.L., Trebicka, J., & Lehmann, J. (2020). Systemic MCP-1 Levels Derive Mainly From Injured Liver and Are Associated With Complications in Cirrhosis. *Frontiers in immunology, 11*:354.
39. Thulin, P., Hornby, R.J., Auli, M., Nordahl, G., Antoine, D.J., Starkey, Lewis, P., Goldring, C.E., Park, B.K., Prats, N., Glinghammar, B., & Schuppe-Koistinen, I. (2017). A longitudinal assessment of miR-122 and GLDH as biomarkers of drug-induced liver injury in the rat. *Biomarkers : biochemical indicators of exposure, response, and susceptibility to chemicals, 22*(5):461-469.
40. Cheng, Z., Lv, Y., Pang, S., Bai, R., Wang, M., Lin, S., Xu, T., Spalding, D., Habib, N., & Xu, R. (2015). Kallistatin, a new and reliable biomarker for the diagnosis of liver cirrhosis. *Acta pharmaceutica Sinica. B, 5*(3):194-200.
41. Kullak-Ublick, G.A., Andrade, R.J., Merz, M., End, P., Benesic, A., Gerbes, A.L., & Aithal, G.P. (2017). Drug-induced liver injury: recent advances in diagnosis and risk assessment. *Gut, 66*(6):1154-1164.
42. Jochum, C., Beste, M., Sowa, J.P., Farahani, M.S., Penndorf, V., Nadalin, S., Saner, F., Canbay, A., & Gerken, G. (2011). Glutathione-S-transferase subtypes α and π as a tool to predict and monitor graft failure or regeneration in a pilot study of living donor liver transplantation. *European journal of medical research, 16*(1):34-40.
43. Horvatits, T., Drolz, A., Roedl, K., Rutter, K., Ferlitsch, A., Fauler, G., Trauner, M., & Fuhrmann, V. (2017). Serum bile acids as marker for acute decompensation and acute-on-chronic liver failure in patients with non-cholestatic cirrhosis. *Liver international : official journal of the International Association for the Study of the Liver, 37*(2):224-231.
44. Novotny, A.R., Emmanuel, K., Maier, S., Westerholt, A., Weighardt, H., Stadler, J., Bartels, H., Schwaiger, M., Siewert, J.R., Holzmann, B., & Heidecke, C.D. (2007). Cytochrome P450 activity mirrors nitric oxide levels in postoperative sepsis: predictive indicators of lethal outcome. *Surgery, 141*(3):376-384.
45. Liu, L., Lu, J., Ye, C., Lin, L., Zheng, S., Zhang, H., Lan, Q., & Xue, Y. (2018). Serum osteopontin is a predictor of prognosis for HBV-associated acute-on-chronic liver failure. *Biomedical reports, 8*(2):166-171.
46. Majumdar, M., Ratho, R., Chawla, Y., & Singh, M.P. (2013). High levels of circulating HMGB1 as a biomarker of acute liver failure in patients with viral hepatitis E. *Liver international : official journal of the International Association for the Study of the Liver, 33*(9):1341-1348.
47. Barnhill, M.S., Real, M., & Lewis, J.H. (2018). Latest advances in diagnosing and predicting DILI: what was new in 2017? *Expert review of gastroenterology & hepatology, 12*(10):1033-1043.
48. Roderburg, C., Benz, F., Vargas, Cardenas, D., Koch, A., Janssen, J., Vucur, M., Gautheron, J., Schneider, A.T., Koppe, C., Kreggenwinkel, K., Zimmermann, H.W., Luedde, M., Trautwein, C., Tacke, F., & Luedde, T. (2015). Elevated miR-122 serum levels are an independent marker of liver injury in inflammatory diseases. *Liver international : official journal of the International Association for the Study of the Liver, 35*(4):1172-1184.
49. Dragoi, D., Benesic, A., Pichler, G., Kulak, N.A., Bartsch, H.S., & Gerbes, A.L. (2018). Proteomics Analysis of Monocyte-Derived Hepatocyte-Like Cells Identifies Integrin Beta 3 as a Specific Biomarker for Drug-Induced Liver Injury by Diclofenac. *Frontiers in pharmacology, 9*:699.
50. Mölleken, C., Ahrens, M., Schlosser, A., Dietz, J., Eisenacher, M., Meyer, H.E., Schmiegel, W., Holmskov, U., Sarrazin, C., Sorensen, G.L., Sitek, B., & Bracht, T. (2019). Direct-acting antivirals-based therapy decreases hepatic fibrosis serum biomarker microfibrillar-associated protein 4 in hepatitis C patients. *Clinical and molecular hepatology, 25*(1):42-51.
51. Santos, V.N., Leite-Mór, M.M., Kondo, M., Martins, J.R., Nader, H., Lanzoni, V.P., & Parise, E.R. (2005). Serum laminin, type IV collagen and hyaluronan as fibrosis markers in non-alcoholic fatty liver disease. *Braz J Med Biol Res. 38*(5):747-53.
52. Imai, H., Kamei, H., Onishi, Y., Ishizu, Y., Ishigami, M., Goto, H., & Ogura, Y. (2018). Diagnostic Usefulness of APRI and FIB-4 for the Prediction of Liver Fibrosis After Liver Transplantation in Patients Infected with Hepatitis C Virus. *Transplantation proceedings, 50*(5):1431-1436.
53. Fabris, C., Smirne, C., Toniutto, P., Colletta, C., Rapetti, R., Minisini, R., Falleti, E., Leutner, M., & Pirisi, M. (2008). Usefulness of six non-proprietary indirect markers of liver fibrosis in patients with chronic hepatitis C. *Clinical chemistry and laboratory medicine, 46*(2):253-259.
54. Day, J.W., & Rosenberg, W.M. (2018). The enhanced liver fibrosis (ELF) test in diagnosis and management of liver fibrosis. *British journal of hospital medicine (London, England : 2005), 79*(12):694-699.
55. Salkic, N.N., Jovanovic, P., Hauser, G., & Brcic, M. (2014). FibroTest/Fibrosure for significant liver fibrosis and cirrhosis in chronic hepatitis B: a meta-analysis. *The American journal of gastroenterology, 109*(6):796-809.
56. Siddiqui, M.S., Patidar, K.R., Boyett, S., Luketic, V.A., Puri, P., & Sanyal, A.J. (2016). Performance of non-invasive models of fibrosis in predicting mild to moderate fibrosis in patients with non-alcoholic fatty liver disease. *Liver Int. 36*(4):572-9.
57. Younossi, Z.M., Loomba, R., Anstee, Q.M., Rinella, M.E., Bugianesi, E., Marchesini, G., Neuschwander-Tetri, B.A., Serfaty, L., Negro, F., Caldwell, S.H., Ratziu, V., Corey, K.E., Friedman, S.L., Abdelmalek, M.F., Harrison, S.A., Sanyal, A.J., Lavine, J.E., Mathurin, P., Charlton, M.R., Goodman, Z.D., Chalasani, N.P., Kowdley, K.V., George, J., & Lindor, K. (2018). Diagnostic modalities for nonalcoholic fatty liver disease, nonalcoholic steatohepatitis, and associated fibrosis. *Hepatology. 68*(1):349-360.
58. Sumida, Y., Nakajima, A., & Itoh, Y. (2014). Limitations of liver biopsy and non-invasive diagnostic tests for the diagnosis of nonalcoholic fatty liver disease/nonalcoholic steatohepatitis. *World journal of gastroenterology, 20*(2):475-485.
59. Abo El-Khair, S.M., El-Alfy, H.A., Elsamanoudy, A.Z., Elhammady, D., Abd-Elfattah, N., Eldeek, B., & Farid, K. (2020). Development of a novel glycated protein-based fibrosis prediction score for determination of significant liver fibrosis in HCV-infected patients, a preliminary study. *Journal of medical virology, Jun* 19.
60. Li, B.B., Li, D.L., Chen, C., Liu, B.H., Xia, C.Y., Wu, H.J., Wu, C.Q., Ji, G.Q., Liu, S., Ni, W., Yao, D.K., Zeng, Z.Y., Chen, D.G., Qin, B.D., Xin, X., Yan, G.L., Dan, Tang., Liu, H.M., He, J., Yan, H., Zhu, W.J., Yu, H.Y., & Zhu, L. (2016). Potentials of the elevated circulating miR-185 level as a biomarker for early diagnosis of HBV-related liver fibrosis. *Scientific reports, 6*:34157.
61. Fourman, L.T., Stanley, T.L., Zheng, I., Pan, C.S., Feldpausch, M.N., Purdy, J., Aepfelbacher, J., Buckless, C., Tsao, A., Corey, K.E., Chung, R.T., Torriani, M., Kleiner, D.E., Hadigan, C.M., & Grinspoon, S.K. (2021). Clinical Predictors of Liver Fibrosis Presence and Progression in Human Immunodeficiency Virus-Associated Nonalcoholic Fatty Liver Disease. *Clinical infectious diseases : an official publication of the Infectious Diseases Society of America, 72*(12):2087-2094.
62. Trépo, C., Chan, H.L.Y., & Lok, A. (2014). Hepatitis B virus infection. *Lancet (London, England), 384*(9959):2053-2063.
63. Wu, D., Zhang, S., Xie, Z., Chen, E., Rao, Q., Liu, X., Huang, K., Yang, J., Xiao, L., Ji, F., Jiang, Z., Zhao, Y., Ouyang, X., Zhu, D., Dai, X., Hou, Z., Liu, B., Deng, B., Zhou, N., Gao, H., Sun, Z., & Li, L. (2020). Plasminogen as a prognostic biomarker for HBV-related acute-on-chronic liver failure. *J Clin Invest. 130*(4):2069-2080..
64. Zhou, J., Mao, W., Shen, L., & Huang, H. (2019). Plasma D-dimer as a novel biomarker for predicting poor outcomes in HBV-related decompensated cirrhosis. *Medicine, 98*(52):e18527.
65. Duan, L., Yang, Q., Yang, J., Hu, Q., Wang, B., Li, P., & Chen, W. (2018). Identification of serum β-catenin as a biomarker in patients with HBV-related liver diseases. *Journal of translational medicine, 16*(1):265.
66. Xiong, Y., Ye, Y., Li, P., Xiong, Y., Mao, J., Huang, Y., Chen, W., & Wang, B. (2018). Serum NOX2 as a new biomarker candidate for HBV-related disorders. *American journal of translational research, 10*(8):2350-2361.
67. Xu, Z., Liu, L., Pan, X., Wei, K., Wei, M., Liu, L., Yang, H., & Liu, Q. (2015). Serum Golgi protein 73 (GP73) is a diagnostic and prognostic marker of chronic HBV liver disease. *Medicine, 94*(12):e659.
68. Patel, E.U., Cox, A.L., Mehta, S.H., Boon, D., Mullis, C.E., Astemborski, J., Osburn, W.O., Quinn, J., Redd, A.D., Kirk, G.D., Thomas, D.L., Quinn, T.C., & Laeyendecker, O. (2016). Use of Hepatitis C Virus (HCV) Immunoglobulin G Antibody Avidity as a Biomarker to Estimate the Population-Level Incidence of HCV Infection.*J Infect Dis. 214*(3):344-52.
69. Jiao, X., Fan, Z., Chen, H., He, P., Li, Y., Zhang, Q., & Ke, C. (2017). Serum and exosomal miR-122 and miR-199a as a biomarker to predict therapeutic efficacy of hepatitis C patients. *J Med Virol. 89*(9):1597-1605.
70. Kanmura, S., Uto, H., Sato, Y., Kumagai, K., Sasaki, F., Moriuchi, A., Oketani, M., Ido, A., Nagata, K., Hayashi, K., Stuver, S.O., & Tsubouchi, H. (2010). The complement component C3a fragment is a potential biomarker for hepatitis C virus-related hepatocellular carcinoma. *Journal of gastroenterology, 45*(4):459-467.
